# Experiences in the treatment of refractory chylothorax associated with lymphoproliferative disorders

**DOI:** 10.1186/s13023-018-0991-3

**Published:** 2019-01-09

**Authors:** Jana Pospiskova, Lukas Smolej, David Belada, Martin Simkovic, Monika Motyckova, Alice Sykorova, Pavla Stepankova, Pavel Zak

**Affiliations:** 1 4th Department of Internal Medicine – Hematology, University Hospital, Sokolska Street 581, 5005 Hradec Kralove, Czech Republic; 2 Faculty of Medicine in Hradec Králové, Charles University Prague, Hradec Kralove, Czech Republic

**Keywords:** Chylothorax, Non-Hodgkin′s lymphoma, Chronic lymphocytic leukemia, Radiotherapy, Thoracic duct

## Abstract

**Background:**

Chylothorax is a rare condition which can be associated with malignant lymphoproliferative disorders (LPDs). We retrospectively analyzed the results of the conservative treatment of 10 patients with persistent non-traumatic malignant chylothorax.

**Results:**

Conservative treatment lead to a decline of chylothorax after mean of 66 days and consisted of the treatment of the underlying disease and of simultaneous long-term supportive care (drainage of the thoracic cavity, dietary measures and nutrition management). In most cases (80%), chylothorax disappeared only after a successful therapeutic response of the underlying disease. Low-dose radiotherapy had very good effects in two patients.

**Conclusion:**

Conservative treatment of malignant chylothorax can be considered a suitable method. Based on our results, successful treatment of the lymphoproliferative disorder seems to be a very important factor for the disappearance of chylothorax.

## Background

Chylothorax is the accumulation of chyle in the pleural cavity. One of the causes of the disturbed function of the thoracic duct is the direct infiltration or compression by a tumor [1–3]. As a result, chyle leaks and accumulates within the pleural cavity. This complication is rare but it may pose a serious problem. Massive effusion may lead to respiratory difficulties, depending on the amount and rate of refilled liquid. The chyle contains a large quantity of fat and of fat-soluble vitamins, proteins, electrolytes and T lymphocytes. The failure of chyle resorption or chyle loss during evacuation may result in serious malnutrition, immune system dysfunction, dehydration and hypovitaminosis [4]. The aim of this article is to present our experience with the conservative management of non-traumatic malignant chylothorax.

## Patients and methods

Between January 2005 and December 2014, ten patients with malignant chylothorax were diagnosed and treated at our department. In these patients mediastinal lymphadenopathy associated with active lymphoproliferative disease was documented and/or malignant cells in effusion which corresponded to the underlying disease were confirmed by cytological and immunophenotypic techniques. Initially, all of the patients with chylothorax required repeated punctures or drainage because of breathing difficulties. Every patient needed 1 to 6 pleural interventions, usually 3 or 4 per each patient (the median was 3.5). Chest radiograph and/or computed tomography of the chest were performed in all the patients to assess the morphological changes of the thorax and to simultaneously evaluate disease activity. Ultrasound of the pleural cavities was performed before thoracentesis. Chylothorax was suspected due to appearance of a milky or opalescent effusion. Because up to 50% cases of chylothorax do not have this characteristic appearance [1, 5], the diagnosis was always verified or confirmed by biochemical methods. The diagnosis of chylothorax was made when the concentration of triglyceride in pleural fluid was greater than 1.24 mmol/l (110 mg/dl), when the fluid to serum triglyceride ratio was greater than 1, and the fluid to serum cholesterol ratio was less than 1 [6]. Pseudochylothorax was differentiated from chylothorax based on the level of triglycerides in the pleural fluid. The triglyceride concentration less than 0.56 mmol/l (50 mg/dl) and the cholesterol greater than 5.18 mmol/l (200 mg/dl) (cholesterol/triglyceride ratio > 1) indicate pseudochylothorax [1, 7, 8].

Persistent chylothorax was considered when 2 weeks of therapy failed to stop chyle production [1, 9]. Afterwards, the efficacy of the chemotherapy was reviewed and computed tomography of the chest was performed again. The results were used to reconsider the next course of treatment.

## Results

Four of the patients had chronic lymphocytic leukemia / small lymphocytic lymphoma (CLL/SLL), five had follicular lymphoma (FL), and one had non-Hodgkin's peripheral T-cell lymphoma - not otherwise specified (PTCL – NOS). None of the CLL patients developed Richter’s transformation. At the time of diagnosis, the median age of the patients was 66 years (95% confidence interval, 57.5–69.7 years) (Table 1). In 8 of the 10 patients, the daily volume of the drained chyle exceeded 1000 ml at least once during the first 7 days. In 4 of the 10 patients the loss was even greater and amounted to more than 1000 ml in 4 successive days during the initial seven-day period (Table 3).

**Table 1:** List of the patients with malignant chylothorax

Patient	Age (years)	Sex	Diagnosis	Stage	M - L	R - L
1.	55	female	CLL	IV	yes	yes
2.	72	male	CLL	0	yes	yes
3.	65	male	CLL	II	yes	yes
4.	71	male	SLL/CLL	IVA	yes	yes
5.	62	female	FL	IIIB	yes	yes
6.	67	male	FL	IVA	yes	yes
7.	60	male	FL	IVB	yes	no
8.	72	female	FL	IVB	yes	yes
9.	45	male	FL	IVA	yes	yes
10.	67	male	PTCL, NOS	IVB	yes	yes

Age – at the time of diagnosis; stage – clinical stage (CLL – according to Rai; FL, SLL, PTCL, NOS – Ann Arbor classification); M-L – mediastinal lymphadenopathy; R-L – retroperitoneal lymphadenopathy

**Fig. 1:**
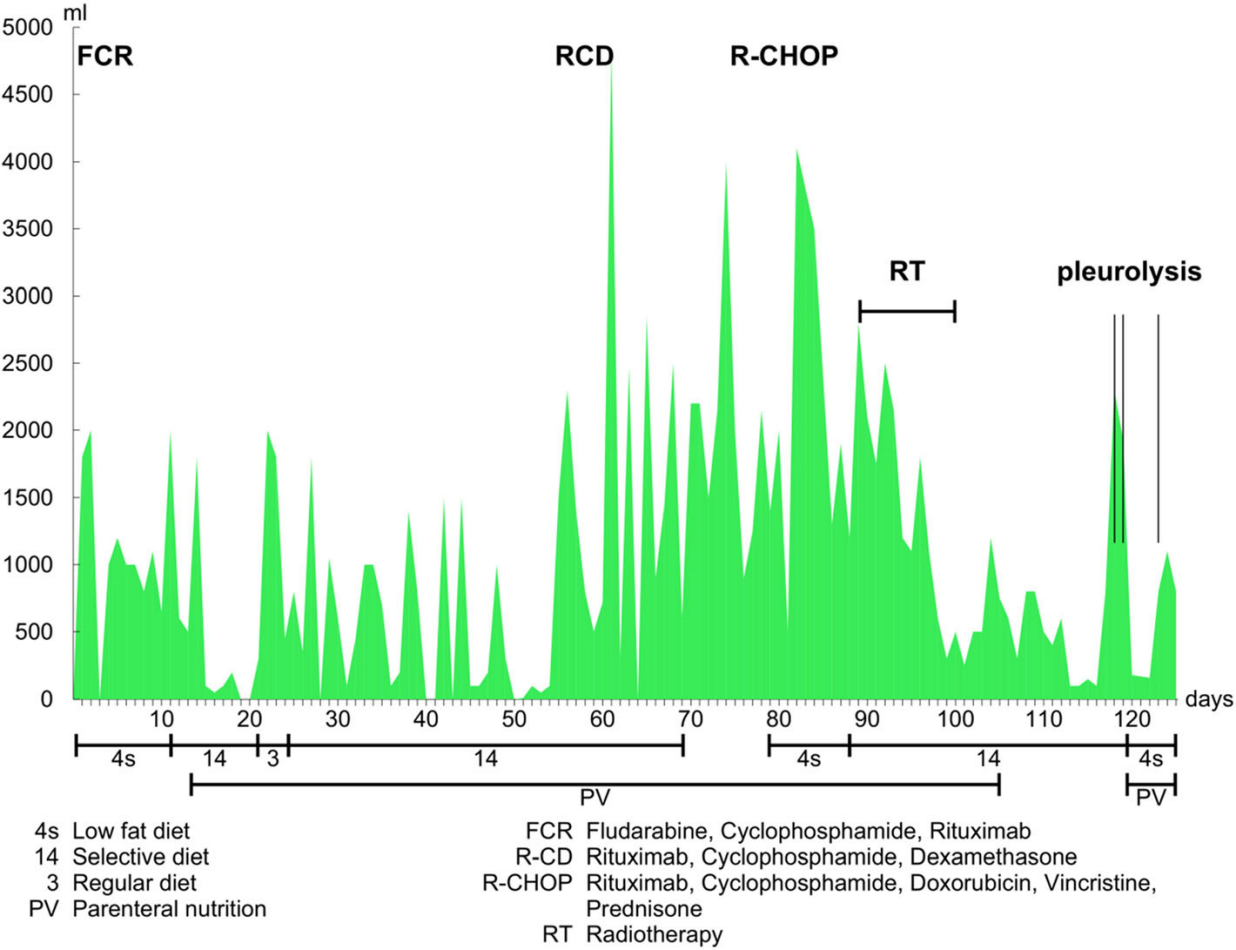
Dependency of drained chylous fluid volume on supportive and effective anticancer treatment. 4 s Low fat diet. FCR: fludarabine, cyclophosphamide, rituximab. 14 Selection diet. RCD: rituximab, cyclophosphamide, high-dose dexamethasone. 3 Regular diet. R-CHOP: rituximab, cyclophosphamide, doxorubicin, vincristine and prednisone. PN Parenteral nutrition. RT Radiotherapy

All patients were started on antineoplastic therapy depending on the type of lymphoproliferation (Table 2). Simultaneously, the conservative treatment of chylothorax which involves the puncture and drainage of chyle was initiated. Eight patients received complete parenteral nutrition with the required levels of liquid and minerals. Seven patients were on a low-fat diet; 3 patients refused this diet. Two patients were hospitalized only briefly to undergo drainage of the chylous effusion and to receive chemotherapy. They did not show any signs of malnutrition and were treated mainly in an out-patient setting. Therefore, complete parenteral nutrition was not necessary. Reduced chyle production improves the spontaneous healing of the lymphatic system. A low-fat diet is recommended to reduce chyle production. This diet may contain medium chain triglycerides (MCT) which are absorbed directly into the bloodstream through the portal vein (Table 3). The local application of streptokinase with premedication of hydrocortisone was used in 3 patients in order to disturb the pleural adhesions. They already had chest tube for drainage of chylous effusion. None of them had empyema. All of these patients had a protracted course of the disease accompanied by several infectious complications and the formation of adhesions. Management of combination chemotherapy regimen for each patient is summarized in Table 2. In one patient with CLL prolonged and significant secretions from the chest drains continued despite a reduced-fat diet and the suppression of CLL activity, the thoracic duct was irradiated with a total dose of 9 Gy in 5 fractions. This was a very effective measure which lead to decreased secretion of chyle enabling the removal of the chest drain on the 26th day since the end of the irradiation. The patient has not experienced relapse of CLL or chylothorax since (Fig. 1). The total time of this patient’s treatment of the chylothorax was 125 days. The patient with SLL had a serosanguinous appearance of the pleural effusion but its biochemical characteristics clearly met the chylothorax criteria [1, 7, 8].

**Table 2:** List of the treatments in patients with malignant chylothorax

Patient	Chemotherapy	Low-fat diet	Parenteral nutrition	RT	Chylothorax treatment duration (days)
1.	FCR	yes	yes	no	82
2.	FCR, R-Dex	yes	yes	no	87
3.	FCR, RCD, R-CHOP	yes	yes	yes	125
4.	FCR	no	no	no	18
5.	obinutuzumab+bendamustine	no	yes	no	25
6.	obinutuzumab+bendamustine	yes	yes	no	82
7.	FCR	yes	yes	yes	69
8.	R-CHOP	yes	yes	no	58
9.	R-CHOP/R-megaCHOP, R-ESHAP/R-GDP, 2x ASCT	yes	yes	no	35
10.	GDP	no	no	no	77

*ASCT* autologous stem cell transplantation, *RT* radiotherapy

**Table 3:** Course of chylothorax treatment and chyle loss through drains

Patient	Initial drainage or puncture for dyspnea (yes-no)	Loss greater than 1000 ml/day at least once during the first 7 days	Loss greater than 1000 ml/day in 4 successive days during the first 7 days	Need for drainage after 14 days of treatment
1.	yes	yes	no	no
2.	yes	yes	no	yes
3.	yes	yes	yes	yes
4.	yes	yes	yes	yes
5.	yes	yes	no	no
6.	yes	no	no	no
7.	yes	yes	no	yes
8.	yes	yes	yes	yes
9.	yes	yes	yes	no
10.	yes	no	no	no

One patient with FL suffered chylothorax as part of the first relapse of FL. The patient received a FCR regimen and the subsequent irradiation of para-aortic and mediastinal lymph nodes with a dose of 4 Gy in 2 fractions, which led to the clearance of chylous effusion (Table 2, patient 7) [10, 11]. Two years later, the patient experienced a chylothorax relapse without signs of an active lymphoma. In this patient the second progression of chylothorax was possibly linked to the previous irradiation of para-aortic and mediastinal lymph nodes. The combination of drainage and dietary measures resulted in the clearance of chylous secretion after 69 days.

In all of these patients suppression of chylothorax was successful. The median time of its clearance was 73 days (95% CI 42.5–89.1 days). Ninety percent of the occurrence of chylothorax was linked to the activity of malignant lymphoproliferation accompanied by mediastinal lymphadenopathy and/or the presence of cancer cells in chylous effusion. The effective treatment of the malignant disease usually also resulted in the clearance of chylous secretion [1, 12]. The second progression of chylothorax was unclear in one patient only (10%), which was associated with the completed radiotherapy of para-aortic and mediastinal lymph nodes. The management included the treatment of both the underlying disease and chylothorax with appropriate chemoimmunotherapy regimens (FCR, R-dex, RCD, R-CHOP, GDP, R-GDP, obinutuzumab + bendamustine, R-ESHAP, and R-mega-CHOP. All treatment regimens are summarized in Table 2). All of the patients required an initial pleural puncture/drainage due to respiratory difficulties. To influence the chylous secretion, a low-fat diet was used in 70% of the patients and parenteral nutrition in 80% of the patients. Radiotherapy was used in two patients only (20%). In one case, irradiation to the thoracic duct with a total dose of 9 Gy in 5 fractions was performed with a very good result. In the other patient, the para-aortic and mediastinal lymph nodes were irradiated with 4 Gy in 2 fractions [10, 11]. A long-term low-fat diet was not followed or observed by 4 of the 7 patients (57%) and three patients refused the dietary measures at the beginning of the treatment. Octreotide/somatostatin is used mainly to treat chylothorax in infants and in patients with traumatic chylothorax [13, 14] and it was not included in our treatment options. Surgical or interventional radiologic approaches which have been on the increase recently were not used due to their narrow specialization and limited availability [8].

The course of chyle loss through chest drains due to changes in conservative treatment in the most problematic patient is shown in a chart (Fig. 1, patient 3). This patient was treated for CLL and his chylothorax lasted for 125 days. The prolonged and substantial loss of chyle led to the use of several treatment options with regard to both chylothorax and CLL. The chemotherapeutic regimen was converted from FCR to RCD and subsequently to R-CHOP and dietary measures were altered repeatedly. It was mainly the dietary measures that were poorly tolerated by the patient. Considering the stationary chylous loss after several days of a low-fat diet and nil per os status with simultaneous parenteral nutrition the patient’s requests for a change in diet were accepted to a less strict diet. Eventually, irradiation of the thoracic duct was used. On the 26th day since the end of the irradiation, the drainage could be terminated and the patient was discharged.

## Discussion

Malignant chylothorax represents more than 50% of the cases of non-traumatic chylothorax, with most of them being caused by lymphoproliferative disorders [1]. We assume that in the cases described in this article, the function/integrity of the thoracic duct and the subsequent leakage of lymph or impaired drainage was due to infiltration or external compression by a tumor [3]. The treatment of chylothorax is usually long and depends on the type of chylothorax, its duration, extent of chyle loss, and the overall condition of the patient. It appears that the crucial measures in the treatment of malignant chylothorax include effective cancer therapy [2, 12, 15] and supplementary interventions like parenteral nutrition, the management of concomitant infectious complications as well as rehabilitation [8].

The shortest and the longest activity of chylothorax in our cohort were 18 and 125 days, respectively. The length of the treatment was evaluated with regard to the necessity to perform puncture/drainage due to respiratory difficulties. A small amount of non-refilling fluid in pleural cavities was often persistent even many weeks after treatment but it did not affect the patients’ everyday life. Therefore, we considered the chylothorax to be cured when chyle was not refilled.

Teng et al. [2] described the treatment of malignant chylothorax in a 20-year study of 18 patients (1991–2011); in 11 of them malignant chylothorax was caused by a lymphoproliferative disease (61%) and in 7 of them by a solid tumor (39%). Indolent lymphomas occurred more often (7 follicular lymphomas, 1 mantle cell lymphoma, 1 lymphoplasmacytic lymphoma) than aggressive lymphomas (1 diffuse large B-cell lymphoma, 1 lymphoblastic lymphoma). In our cohort of 10 patients with lymphoproliferative diseases observed for a period of 9 years (2005–2014), indolent lymphoproliferative disorders (5 follicular lymphomas, 4 chronic lymphocytic leukemia/small lymphocytic lymphomas) were also more frequent than aggressive lymphomas (1 non-Hodgkin peripheral T-cell lymphoma not otherwise specified). Compared to Teng et al., our experience also implies that the main factor of the treatment of malignant chylothorax is whether anticancer therapy is effective. Chylothorax cleared in most patients (80%) only after reaching response of the hemato-oncological disease which had caused thoracic duct damage. In the cohort presented by Teng et al. chylothorax cleared simultaneously with establishing the complete remission of lymphoproliferation in 6 out of 11 patients (55%).

Doerr et al. of Mayo Clinic [16] analyzed the etiology of chylothorax in a study of 203 patients over 20 years (January 1980 – December 2000). Malignant chylothorax was reported only in 34 patients (16.7%), 23 of them were patients with a lymphoma (11.3%, 20 had non-Hodgkin′s lymphoma and 3 had Hodgkin lymphoma), 5 had chronic lymphocytic leukemia and 6 suffered from another malignity (5 had metastatic disease and 1 had lung carcinoma). Like in our cohort, Doerr et al. observed that chylothorax tended to occur in an advanced lymphoma.

Another widely debated issue is the necessity to follow dietary measures. 60–70% of absorbed fats get into the bloodstream through the thoracic duct [9]. The disturbed integrity of the duct wall leads to massive nutritional loss which is most often compensated by the administration of complete parenteral nutrition which provides an additional benefit, i.e. reduced flow of chyle, thus improving the spontaneous healing of the lymphatic system. A low-fat diet is recommended to reduce chyle production. Paradoxically, the shortest treatment period was required by a patient who did not have this dietary measure. This suggests that the crucial role is played by the effective treatment of underlying disease [2, 12]. However, the small number of patiens in our cohort does not allow unambiguous conclusions. In addition, the extent of lymphadenopathy does not correlate with the chylothorax treatment period in our cohort. For instance, a female patient with a 16 cm retroperitoneal lymphadenopathy was hospitalized for 58 days while a male patient with a small mediastinal and retroperitoneal lymphadenopathy (2 cm) needed 87 days of hospitalization. Generally, advanced stages of the disease in which chylothorax occurred obviously prevailed (Table 1) [3, 16].

For the management of recurrent pleural effusions, an indwelling pleural catheter (IPC) is successfully used [17–19]. Its usefulness is also in the treatment of other conditions including chylothorax [17–19]. The IPC is a tunneled pleural catheter with advantage of allowing patients to be managed in the outpatient setting [19]. Other interventions were chosen in our cohort because of an expected response to adequate treatment and non-palliative management of the underlying disease.

According to the literature, the reduction of chyle production was supported by the administration of somatostatin or octerotide, especially when chylothorax was treated in infants and in patients with traumatic chylothorax [13, 14, 20, 21]. In the case of persistent, so-called ‘high volume’ chylothorax with a daily chyle loss of more than 1000 ml lasting 5 days [8, 22], conversion from conservative to surgical treatment is recommended, most often by ligating the thoracic duct [1, 8, 9, 22] which could be combined with pleurodesis [23]. One of the less frequent approaches applied to non-traumatic tumor-associated chylothorax, is the irradiation of the thoracic duct which was described by Gerstein et al. [15]. Fractional irradiation of the celiac trunk and of the thoracic duct with a dose of 20.4 Gy and 15 Gy, respectively, resulted in the complete remission of chylothorax in a patient with mediastinal lymphadenopathy that accompanied the lymphoma. The major decrease in drained waste was observed already at a dose as low as 7.5 Gy [24]. In our cohort, effective doses in the two patients were 9 Gy and 4 Gy [10, 11]. The first patient had CLL with a complicated course of disease (The loss of chyle through thoracic drains during changes in therapeutic regimens is demonstrated in chart No. 1, patient 3.). It is not self-evident whether the improvement of the patient’s condition can be attributed to the effect of the treatment of CLL or to the irradiation of the thoracic duct, yet the irradiation of the thoracic duct seems to be a gentle and effective treatment option in persistent chylothorax accompanying malignant lymphoproliferation. Moreover, in our study irradiation was provided in several small doses which enabled further treatment in the future if required.

Radiological approaches have recently become widely used in the treatment of chylothorax. Contrast lymphangiography facilitates a good visualization of the lymphatic system and the chyle leakage site. Furthermore, it is known that contrast lymphangiography also has therapeutic effects as chylothorax becomes suppressed after its application in up to 89% of the patients [25]. Cope et al. describe a low-risk percutaneous approach for the embolization of the thoracic duct via the *cisterna chyli* or retroperitoneal lymphatic vessels [24]. Various modifications of transcutaneous embolization approaches are provided by selected centers only [8, 26–30]. Alternative transport procedures indicated in clearly defined situations include introduction of a pleuroperitoneal or pleurovenous shunt, establishing an anastomosis between the thoracic duct and the azygos vein, or transjugular shunt [16, 31–33].

## Conclusion

Based on our experience the crucial factor affecting the clearance of chylothorax appears to consist in suppressing the activity of underlying hematological disease. The median duration of conservatively managed chylothorax in our patients was 73 days. This type of treatment is rather long and demanding and requires patience on the part of both the patient and the medical team. Nevertheless, with the sensitivity of underlying hematological disease to treatment, the general prognosis of the patient is quite good. Our cohort displayed a low risk of recurrence (20%) which was linked to relapsed lymphoma only in some of the patients. Low-dose irradiation presents an interesting treatment option for refractory chylothorax, which was proven in two patients in the cohort. The future diagnostics and treatment are likely to benefit especially from invasive radiological methods.

## Abbreviations

CLL: Chronic lymphocytic leukemia
FCR: Fludarabine, cyclophosphamide, rituximab
FL: Follicular lymphoma
LPDs: Lymphoproliferative disorders
MCT: Medium chain triglycerides
M-L: Mediastinal lymphadenopathy
PBSCT: Peripheral blood stem cell transplantation
PTCL-NOS: Peripheral T-cell lymphoma - not otherwise specified
PV: Parenteral nutrition
RCD: Rituximab, cyclophosphamide, high-dose dexamethasone
R-CHOP: Rituximab, cyclophosphamide, doxorubicin, vincristine, and prednisone
R-dex: Rituximab, high-dose dexamethasone
R-ESHAP: Rituximab, etoposide, methylprednisolone, cytarabine, platinium
R-GDP: Rituximab, gemcitabine, dexamethasone, cisplatin
R-L: Retroperitoneal lymphadenopathy
RT: Radiotherapy
SLL: Small lymphocytic lymphoma
